# Mechanism of Negative Thermal Expansion in Monoclinic Cu_2_P_2_O_7_ from First Principles

**DOI:** 10.1021/acs.jpclett.3c02856

**Published:** 2023-12-27

**Authors:** Yasuhide Mochizuki, Kaede Nagamatsu, Hiroki Koiso, Toshihiro Isobe, Akira Nakajima

**Affiliations:** Department of Materials Science and Engineering, School of Materials and Chemical Technology, Tokyo Institute of Technology, Tokyo 152-8550, Japan

## Abstract

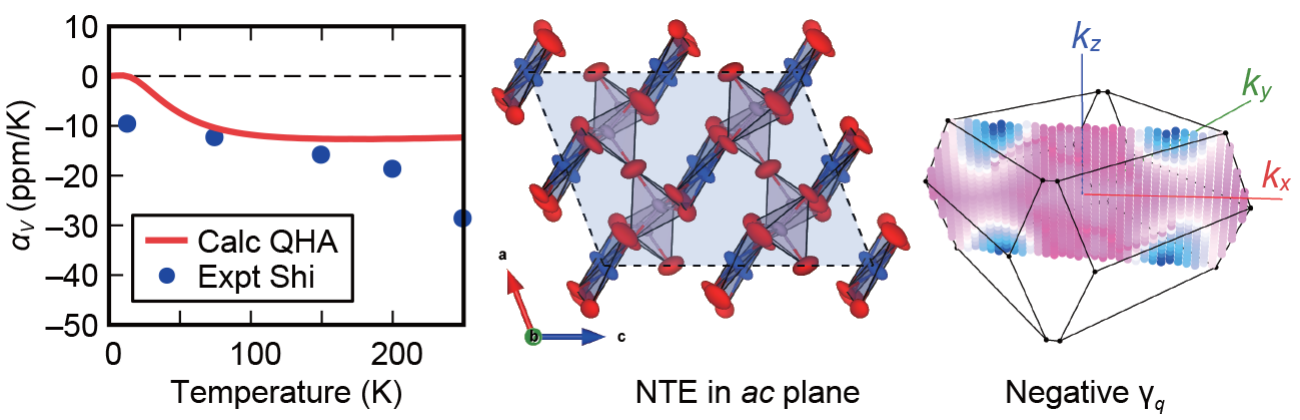

Negative thermal expansion (NTE) materials generally have high-symmetry space groups, large average atomic volumes, and corner-sharing octahedral and tetrahedral coordination structures. By contrast, monoclinic α-Cu_2_P_2_O_7_, which has a small average atomic volume and edge-sharing structure, has been reported to exhibit NTE, the detailed mechanism of which is unclear. In this study, we investigate the *A*_2_*B*_2_O_7_ polymorphs and analyze the NTE behavior of α-Cu_2_P_2_O_7_ using first-principles lattice-dynamics calculations. From the polymorphism investigation in 20 *A*_2_*B*_2_O_7_ compounds using 6 representative crystal structures, small *A* and *B* cationic radii are found to stabilize the α-Cu_2_P_2_O_7_-type structure. We then analyze the NTE behavior of α-Cu_2_P_2_O_7_ using quasi-harmonic approximation. Our calculated thermal expansion coefficients and anisotropic atomic displacement parameters were in good agreement with those of the experimental reports at low temperatures. From the mode-Grüneisen parameter distribution plotted over the entire first-Brillouin zone, we found that the phonon contributing most significantly to NTE emerges not into the special points but between them. In this phonon mode, the O connecting two PO_4_ tetrahedra rotates, and the Cu and O vibrate perpendicular to the bottom of the CuO_5_ pyramidal unit, which folds the *ac* lattice plane. This vibration behavior can explain the experimentally reported anisotropic NTE behavior of α-Cu_2_P_2_O_7_. Our results demonstrate that the most negative mode-Grüneisen parameter contributing to NTE behavior is not always located on high-symmetry special points, indicating the importance of lattice vibration analyses for the entire first-Brillouin zone.

Negative thermal expansion (NTE) is an intriguing and counterintuitive physical phenomenon in which volume shrinks as temperature increases. Previously reported prototypical NTE materials include ZrW_2_O_8_ (*P*2_1_3, *Pa*3̅), ZrV_2_O_7_ (*Pa*3̅), Y_2_W_3_O_12_ (*Pbcn*), NbVO_5_ (*Pnma*), KZr_2_P_3_O_12_ (*R*3̅*c*), and ReO_3_ (*Pm*3̅*m*).^1−7^ The common features of these NTE materials are (i) being composed of point-sharing octahedral and tetrahedral framework structures, (ii) having high-symmetry space groups, and (iii) having large average atomic volumes (AAVs).^8−11^ Generally, it has been reported that a large AAV tends to yield large NTE behavior;^11^ the critical point of the AAV that gives rise to NTE behavior is reported to be 16 Å^3^. Contrary to this general trend, monoclinic α-Cu_2_P_2_O_7_ with *C*2/*c* symmetry and a small AAV (10.97 Å^3^ at 300 K) has been experimentally reported to exhibit NTE below 350 K.^12^ The crystal structure of Cu_2_P_2_O_7_ is interweaved with distorted edge- and corner-shared pyramidal CuO_5_ units and corner-shared tetrahedral PO_4_ units. In addition, α-Cu_2_P_2_O_7_ exhibits large NTE behavior, with a volume thermal expansion coefficient α_*V*_ of −27.69 ppm/K, shrinking in the *a*- and *c*-axis directions (α_*a*_ = −30.11 ppm/K, and α_*c*_ = −10.75 ppm/K) and slightly expanding in the *b*-axis direction (α_*b*_ = 3.45 ppm/K) upon heating.^12^ Because the characteristics of Cu_2_P_2_O_7_ are different from those of the prototypical NTE materials, the origin of the NTE behavior of α-Cu_2_P_2_O_7_ is not fully clarified. Therefore, mechanism elucidation of the NTE behavior and investigation of the polymorphism of Cu_2_P_2_O_7_, which is unique among the NTE materials, may lead to not only an understanding of NTE behavior but also the exploration of new NTE materials.

In this study, we investigate the polymorph and analyze the NTE behavior of α-Cu_2_P_2_O_7_ using the first-principles lattice-dynamics calculations. In the first half, we compare the polymorphism of 6 representative crystal structures of *A*_2_*B*_2_O_7_ (*A* = Cu, Zn, Mg, Sr, Sc, or Y; *B* = Ge, Sn, Ti, Zr, P, V, or Ta) and indicate that only α-Cu_2_P_2_O_7_ structures (the *C*2/*c* phase) of Cu_2_P_2_O_7_, Cu_2_V_2_O_7_, and Zn_2_V_2_O_7_ are found to be dynamically stable. In the latter half, we discuss the NTE mechanism for α-Cu_2_P_2_O_7_ in detail. The distribution of the mode-Grüneisen parameters over the entire first-Brillouin zone is illustrated, and it is shown that the phonon modes that contribute most significantly to the NTE behavior are located between the special points, not on the special points.

The first-principles calculations were performed using the projector-augmented-wave (PAW) method^13^ as implemented in VASP.^14,15^ For all of the calculations, the GGA-PBEsol functional^16^ was adopted (see Table S1 for the functional dependencies of lattice constants), and the plane-wave cutoff energy was set to 550 eV. The cutoff radii and valence electronic configurations of the PAW data sets are listed in Table S2. The initial crystal structures used in this study were extracted from Materials Project.^17^ For the calculations of crystal polymorphs, Monkhorst-pack *k*-point meshes of 5 × 5 × 5, 8 × 8 × 9, 6 × 6 × 3, 5 × 5 × 5, 3 × 3 × 7, and 7 × 7 × 4 were employed for the *Fd*3̅*m*, *C*2/*m*, *P*4_3_2_1_2, *Imma*, *Cmcm*, and *C*2/*c* phases, respectively. The phonon frequencies were derived from the calculated force constant using PHONOPY.^18,19^ In the analysis of the NTE behavior of α-Cu_2_P_2_O_7_, to calculate the force constants, we used the 2 × 2 × 2 supercells, which were constructed by expanding the relevant conventional cells. In the analyses within the quasi-harmonic approximation (QHA),^20^ the volume thermal expansion coefficients and Grüneisen parameters were calculated by isotropically changing lattice parameters *a*, *b*, and, *c* from relaxed lattice parameters *a*_0_, *b*_0_, and *c*_0_, respectively, in the range of −0.66% to 0.66% in increments of 0.33%. The anisotropic atomic displacement parameters and thermal ellipsoids were calculated using PHONOPY.^21,22^ The chemical bonding analyses through crystal orbital Hamilton populations (COHPs) were performed using LOBSTER.^23,24^ Projected electronic DOSs were extracted using VASPKIT.^25^ Moreover, the colinear antiferromagnetic configuration (see Figure S1) in Cu_2_P_2_O_7_ was used, which was also mentioned in the previous studies.^26,27^ Note that we did not adopt the +*U* correction to the *d* electrons in the Cu^2+^ ions (see section 4 of the Supporting Information for the discussion of the +*U* correction).

To clarify which compounds could be stabilized into the *C*2/*c* phase (α-Cu_2_P_2_O_7_ structure), we investigated the *A*_2_*B*_2_O_7_ polymorphs. At first, 595 *A*_2_*B*_2_O_7_ oxides were extracted from Materials Project.^17^ These structures were then classified according to their structural features using PYMATGEN,^28^ and the following 6 representative crystal structures were derived: the *Fd*3̅*m* (pyrochlore structure), *C*2/*m*, *P*4_3_2_1_2, *Imma*, *Cmcm*, and *C*2/*c* phases (illustrated in Figure 1). Here, we considered 20 *A*_2_*B*_2_O_7_ compounds by using the extracted prototypes. The *A* and *B* sites are composed of (i) a combination of trivalent early transition metals *A*^3+^ (Sc or Y) and tetravalent post or early transition metals *B*^4+^ (Ge, Sn, Ti, or Zr), (ii) a combination of divalent alkaline earth metals *A*^2+^ (Mg or Sr) and pentavalent phosphorus or early transition metals *B*^5+^ (P, V, or Ta), and (iii) a combination of late transition metals *A*^2+^ (Cu or Zn) and pentavalent phosphorus or early transition metals *B*^5+^ (P, V, or Ta). As for Zn_2_P_2_O_7_, Sr_2_P_2_O_7_, Sr_2_V_2_O_7_, and Mg_2_V_2_O_7_, because their experimentally reported ground-state structures listed in Materials Project^17^ were not included in the 6 prototypes, we additionally calculated the relevant ground-state structures, that is, the *Pbcm*, *Pnma*, *P*4_1_, and *P*1̅ phases.

**Figure 1:**
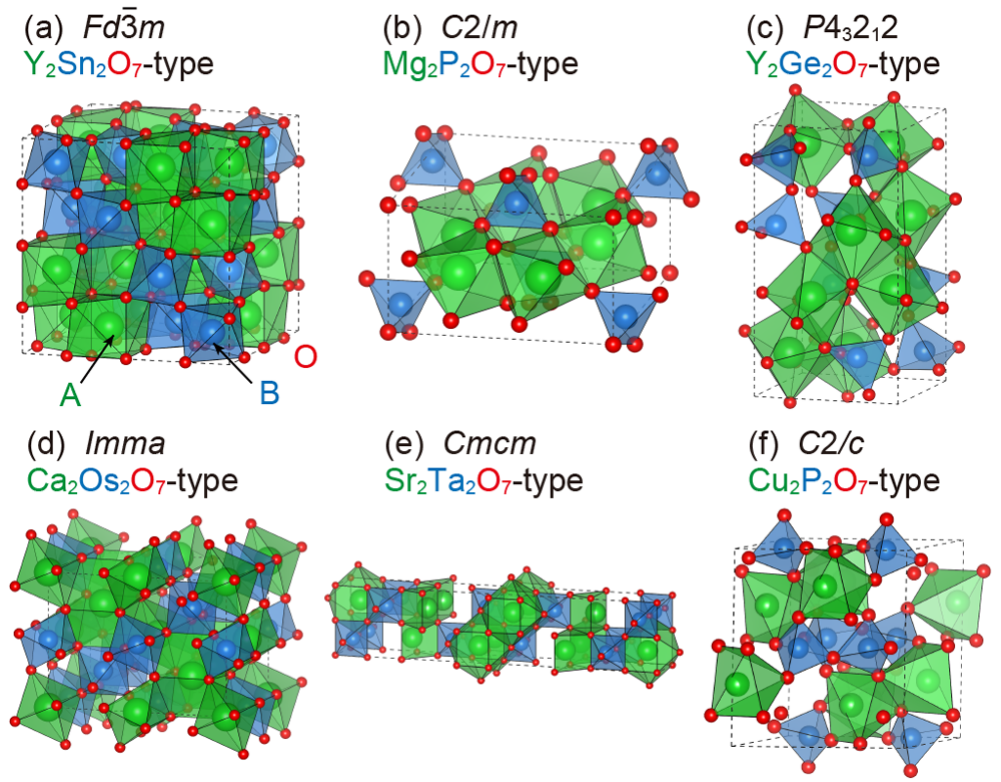
Crystal structures of (a) Y_2_Sn_2_O_7_-type *Fd*3̅*m*, (b) Mg_2_P_2_O_7_-type *C*2/*m*, (c) Y_2_Ge_2_O_7_-type *P*4_3_2_1_2, (d) Ca_2_Os_2_O_7_-type *Imma*, (e) Sr_2_Ta_2_O_7_-type *Cmcm*, and (f) Cu_2_P_2_O_7_-type *C*2/*c* phases. The green, blue, and red elements represent the *A*-, *B*-, and O-site atoms, respectively, in *A*_2_*B*_2_O_7_ compounds.

The results of the total energy comparison for the polymorphs are presented in Figure 2. Here, we calculated the relative total energies with respect to the most stable phases among the 6 or 7 polymorphs (see section 5 of the Supporting Information for details of the determination of the most stable phase). As shown in Figure 2, the *C*2/*c* phase is the most stable in the 7 compounds: Sc_2_Zr_2_O_7_, Mg_2_Ta_2_O_7_, Cu_2_P_2_O_7_, Cu_2_V_2_O_7_, Cu_2_Ta_2_O_7_, Zn_2_V_2_O_7_, and Zn_2_Ta_2_O_7_ (see the red squares). Moreover, we examined whether these compounds within the *C*2/*c* phase are dynamically stable by calculating their phonon bands. We found that Cu_2_P_2_O_7_, Cu_2_V_2_O_7_, and Zn_2_V_2_O_7_ are dynamically stable whereas the others (Sc_2_Zr_2_O_7_, Mg_2_Ta_2_O_7_, Cu_2_Ta_2_O_7_, and Zn_2_Ta_2_O_7_) are dynamically unstable (see Figure S4 for their phonon bands).

**Figure 2:**
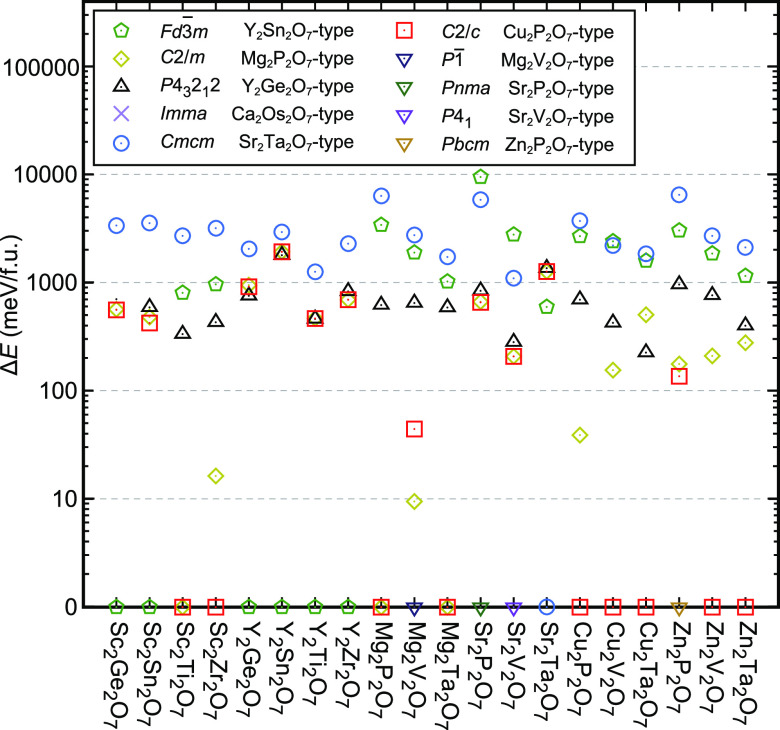
Total energies for *A*_2_*B*_2_O_7_ compounds with respect to that of the most stable phase among the polymorphs on a log scale.

Next, we investigated the correlation between the ionic radius ratio and the structural stability of the most stable phases. The ionic radius ratio of cations *A* and O (*r*_*A*_/*r*_O_) and that of cations *B* and O (*r*_*B*_/*r*_O_) were set as the horizontal and vertical axes, respectively, in the map of structural stability (Figure 3). The ionic radii of *A* and *B* for the 20 compounds were estimated from Shannon’s ionic radius^29^ by considering the effective coordination numbers in the most stable phase among the polymorphs (see section 6 of the Supporting Information and Figure S5). In the map of structural stability, the *C*2/*c* phases, which have 5-coordinated *A* cations and 4-coordinated *B* cations, are distributed in the ranges of *r*_*A*_/*r*_O_ ≤ 0.732 and *r*_*B*_/*r*_O_ ≤ 0.414. This trend can be understood by Pauling’s first law, indicating that the coordination number of a cation is determined by the ionic radius ratio of the cation and the anion. Specifically, Cu_2_P_2_O_7_, Cu_2_V_2_O_7_, and Zn_2_V_2_O_7_ are located in the lower range of *r*_*A*_/*r*_O_ compared to that of Zn_2_P_2_O_7_, Mg_2_P_2_O_7_, and Mg_2_V_2_O_7_. This trend should be attributed to the difference in coordination preference among Cu, Zn, and Mg. It has been reported that the square pyramidal coordination is preferred in the order of Cu^2+^, Zn^2+^, and Mg^2+^.^30^ Cu_2_P_2_O_7_, Cu_2_V_2_O_7_, and Zn_2_V_2_O_7_ (*C*2/*c* phases) have 5-coordinated square pyramidal *A* cations, whereas Zn_2_P_2_O_7_ (*Pbcm*), Mg_2_P_2_O_7_ (*C*2/*m*), and Mg_2_V_2_O_7_ (*P*1̅) do not. Similarly, the *C*2/*c* phases of Sc_2_Zr_2_O_7_, Mg_2_Ta_2_O_7_, Cu_2_Ta_2_O_7_, and Zn_2_Ta_2_O_7_ become dynamically unstable because Zr^4+^ and Ta^5+^ strongly prefer 6-coordinated octahedral structures.^30^ In fact, these 4 compounds are located in the range of *r*_*B*_/*r*_O_ ≥ 0.414, indicating that Zr^4+^ and Ta^5+^ are too large to be located in the center of a 4-coordinated tetrahedron.

**Figure 3:**
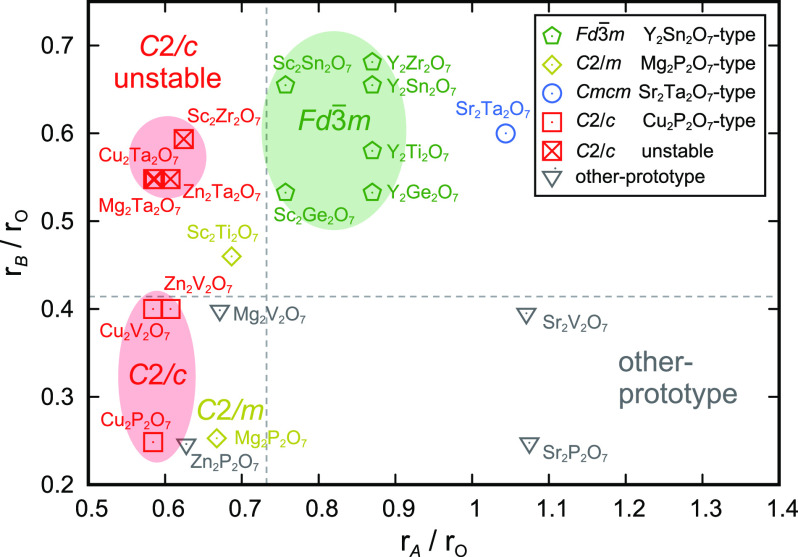
Map of structural stability aligned with ionic radius ratios *r*_*A*_/*r*_O_ and *r*_*B*_/*r*_O_. The pentagons, diamonds, circle, squares, and triangles represent the *Fd*3̅*m*, *C*2/*m*, *Cmcm*, *C*2/*c*, and other prototype phases, respectively. The crossed squares represent unstable phases.

Combining all of the discussion using the map of stability, we can also see that the *C*2/*c* phase of α-Cu_2_P_2_O_7_ is relatively rare because 5-coordinated square pyramidal and 4-coordinated tetrahedral cations are essential to realizing the α-Cu_2_P_2_O_7_ structure. In other words, to form the *C*2/*c* phase, only small cations can be adopted. It is worth noting that the small coordination number of the *C*2/*c* phases seemingly decreases the AAV. Indeed, only Cu_2_P_2_O_7_, Cu_2_V_2_O_7_, and Zn_2_V_2_O_7_, which have been experimentally reported to exhibit framework-type (phonon-induced) NTE,^12,31−33^ were found to be dynamically stable in this study.

Hereafter, we discuss the NTE behavior of Cu_2_P_2_O_7_ and its underlying mechanism. Figure 4 presents the temperature dependence of the volume thermal expansion coefficients α_*V*_ calculated within QHA. The calculated α_*V*_ are in good agreement with the experimental reports below 200 K, whereas they are not above 250 K. These results imply that the NTE behavior of Cu_2_P_2_O_7_ below 200 K can be explained by phonon-induced NTE, which is known as the tension effect.^10,34^ On the contrary, the inconsistency of α_*V*_ above 250 K would be attributed to the effect of thermal fluctuations associated with the phase transition at around 350 K. The NTE behavior of Cu_2_P_2_O_7_ around the phase transition temperature is analogous to that of Zn_2_V_2_O_7_, Zn_2_P_2_O_7_, and Mg_2_P_2_O_7_.^33,35,36^

**Figure 4:**
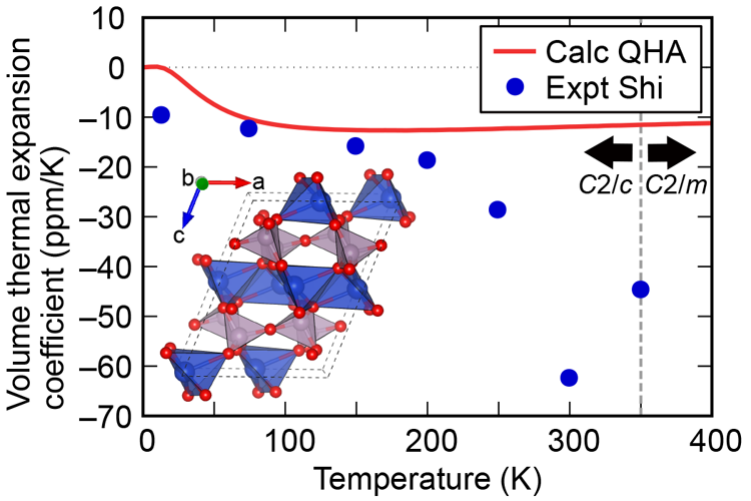
Calculated volume thermal expansion coefficients via QHA for α-Cu_2_P_2_O_7_ as a function of temperature (red line). The experimental values from ref (12) are also indicated (blue circles). The crystal structure of α-Cu_2_P_2_O_7_ (the *C*2/*c* phase) is also illustrated.

To clarify which phonons mainly contribute to the NTE nature, we calculated the mode-Grüneisen parameters γ_***q***_ [=–*V*/ω_***q***_(∂ω_***q***_/∂*V*)_*T*_^37^] at wave vector ***q***, which is regarded as the degree of anharmonicity owing to phonon frequency variation by the volume change at a constant temperature (Figure 5; see section 7 of the Supporting Information and Table S3 for the validity of the calculated phonon frequency). In Figure 5a, the phonon modes colored with blue indicate negative mode-Grüneisen parameters, implying that those phonons contribute mainly to the NTE behavior, which are densely located in the low-frequency range below 5 THz. Figure 5b shows a phonon mode with robustly negative mode-Grüneisen parameters between the H and Z points. From these results, we can expect that the phonons with the most negative mode-Grüneisen parameter might not exist on the special points of the *C*2/*c* space group, and hence, the mode-Grüneisen parameters for the entire first-Brillouin zone were then investigated (Figure 6). The Grüneisen parameter is found to be the most negative at reciprocal point (^1^/_4_, ^1^/_4_, ^1^/_4_), not on the special points. In other words, the most significant phonon mode for the NTE behavior of α-Cu_2_P_2_O_7_ emerges from (^1^/_4_, ^1^/_4_, ^1^/_4_), and the cluster of phonons in the vicinity of this point is expected to contribute mainly to the NTE behavior. These results suggest the importance of considering phonon modes not only on the band paths but also in the entire first-Brillouin zone. Recently, Dove et al. reported that the NTE behavior of ScF_3_ is attributed to not only the phonons at the R and M points but also the phonons between the R and M points,^38^ which also showed the importance of phonon observation in the whole first-Brillouin zone. Additionally, there are several ways to adopt band paths,^39−41^ which affect the physical properties. For instance, it has been reported that the band gap of GePtS cannot be properly evaluated when the band path is not adopted properly because the valence band maximum (VBM) and conduction band minimum (CBM) change depending on the band path.^41^ Similarly, in the case presented here, the appearance of the mode-Grüneisen parameter changes depending on the band path, and thus, the phonon band analyses on a particular band path may miss the phonon modes that are important for the NTE mechanism (see Figure S6).

**Figure 5:**
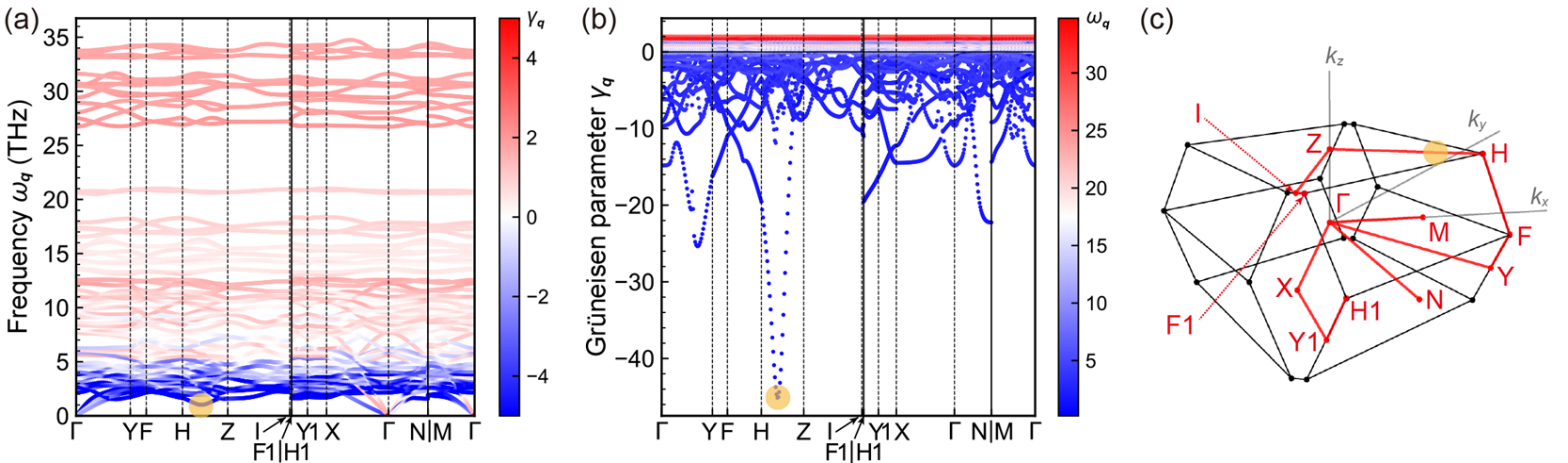
(a) Phonon band with the mode-Grüneisen parameters for α-Cu_2_P_2_O_7_. Blue and red indicate negative and positive mode-Grüneisen parameters, respectively. (b) Mode-Grüneisen parameters along the path of the first-Brillouin zone of the *C*2/*c* space group, which are colored according to the calculated phonon frequency. (c) First-Brillouin zone shape and paths used in the phonon dispersion curves. The special reciprocal points are located at Γ(0, 0, 0), Y(0.44, 0.44, −0.68), F(0.48, 0.48, −0.48), H(0.4, 0.4, 0.12), Z(0, 0, 0.5), I(0.5, −0.5, 0.5), F1(0.52, −0.48, 0.48), H1(0.6, −0.4, −0.12), Y1(0.56, −0.44, −0.32), X(0.5, −0.5, 0), N(0.5, 0, −0.5), and M(0.5, 0, 0).

**Figure 6:**
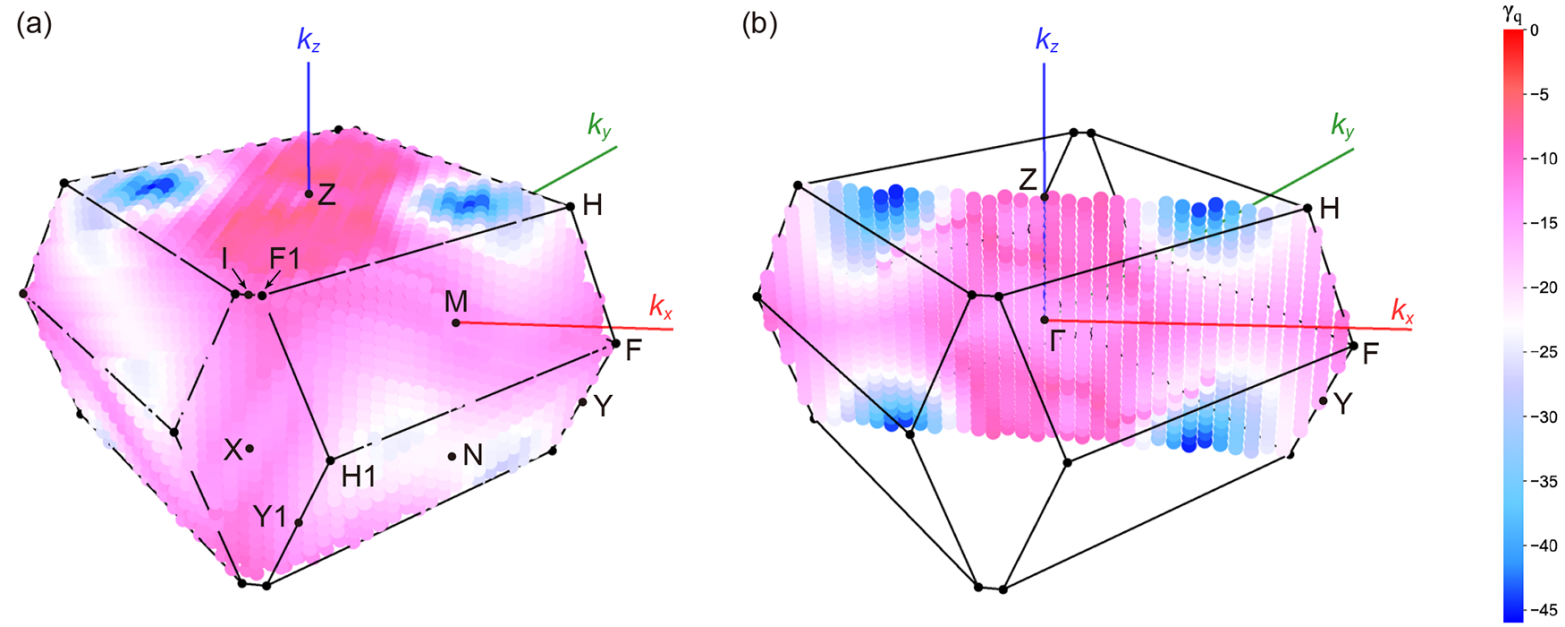
Distribution of the lowest values of the mode-Grüneisen parameters for α-Cu_2_P_2_O_7_ at relevant wave vector (a) in the whole reciprocal space of space group *C*2/*c* and (b) at the cross section for *k*_*x*_ = *k*_*y*_. The special reciprocal points that are defined in Figure 5c are indicated.

We depict the most essential phonon for the NTE behavior located at (^1^/_4_, ^1^/_4_, ^1^/_4_) in Figure 7. In this mode, the oxygen (O1) that connects the two PO_4_ tetrahedral units rotates around the P–O1–P bond, and the copper and oxygen (O4) vibrate perpendicular to the CuO_4_ plane (the bottom of the CuO_5_ pyramidal unit) (see Movie S1). In other words, the vibrations at (^1^/_4_, ^1^/_4_, ^1^/_4_) fold the *ac* lattice plane. From this mode, we can catch a glimpse of the origin of NTE in the *ac* lattice plane for α-Cu_2_P_2_O_7_: (i) the effect of the CuO_4_ quadrilaterals coming closer together as O1 rotates around the *a*-axis direction and (ii) the CuO_4_ quadrilaterals that are folded into a bellows-like shape as Cu and O vibrate perpendicular to the CuO_4_ planar structures. These mechanisms do not conflict with the experimentally reported contractions in the *a*- and *c*-axis directions and expansion in the *b*-axis direction upon heating. It is noteworthy that the phonon mode in Cu_2_P_2_O_7_ with the most negative mode-Grüneisen parameter at (^1^/_4_, ^1^/_4_, ^1^/_4_) is different from the rigid unit modes, which can be observed in ScF_3_^10,38^ and β-cristobalite SiO_2_.^10^ In addition, d’Ambrumenil et al.^42^ reported that the NTE of ZnNi(CN)_4_ stems from the transverse motion of Ni in the direction perpendicular to its square planar environment. The NTE behavior of Cu_2_P_2_O_7_ is analogous to that of ZnNi(CN)_4_ because both units of CuO_4_ quadrilaterals in Cu_2_P_2_O_7_ (bottom of the CuO_5_ pyramidal unit) and NiC_4_ squares in ZnNi(CN)_4_ can be regarded as a two-dimensional local environment.

**Figure 7:**
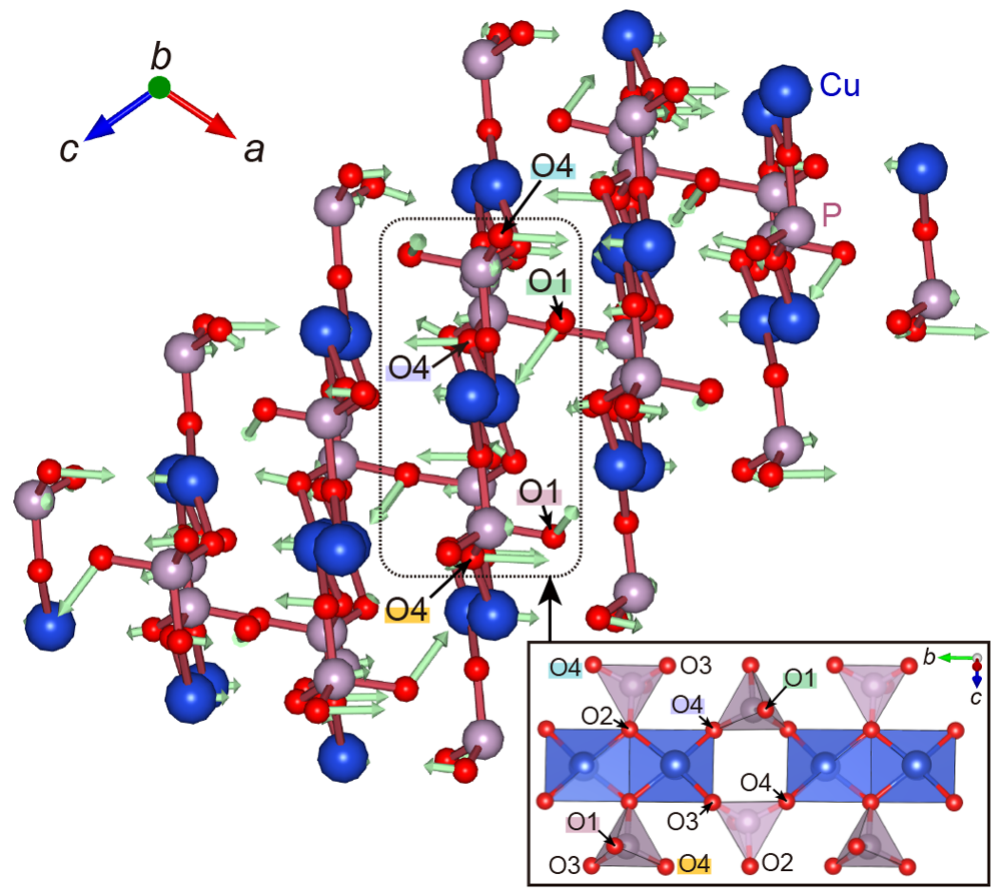
Visualized phonon with the lowest frequency at (^1^/_4_, ^1^/_4_, ^1^/_4_), which has the most negative mode-Grüneisen parameter. The bottom right inset shows a side view of the layer composed of CuO_4_ quadrilaterals.

Here, the looming question is the feature of the phonons with robustly negative mode-Grüneisen parameter γ_***q***_. The phonons with robustly negative γ_***q***_ are located at the lowest frequency concurrent with the zero gradient in the phonon bands (see the yellow circles in Figure 5). One can see that the phonon with the most negative γ_***q***_ is located at the local minimum of ω_***q***_ in the convex downward phonon band, which stems mainly from the inversely proportional relation between γ_***q***_ and ω_***q***_. Indeed, the sign and absolute value of γ_***q***_ are determined by the volume derivative of frequency, (∂ω_***q***_/∂*V*)_*T*_. In addition, the gradient in the phonon bands is identical to the group velocity, and hence, we also investigated the correlation between the group velocities and mode-Grüneisen parameters γ_***q***_. Intriguingly, as shown in Figure 8, the phonon mode in which γ_***q***_ is the most negative has near-zero or low group velocity. In short, the feature of the phonons with a strongly negative γ_***q***_ is the vibration close to a standing wave. This perspective implies that the coexistence of NTE and high thermal conductivity would be challenging.

**Figure 8:**
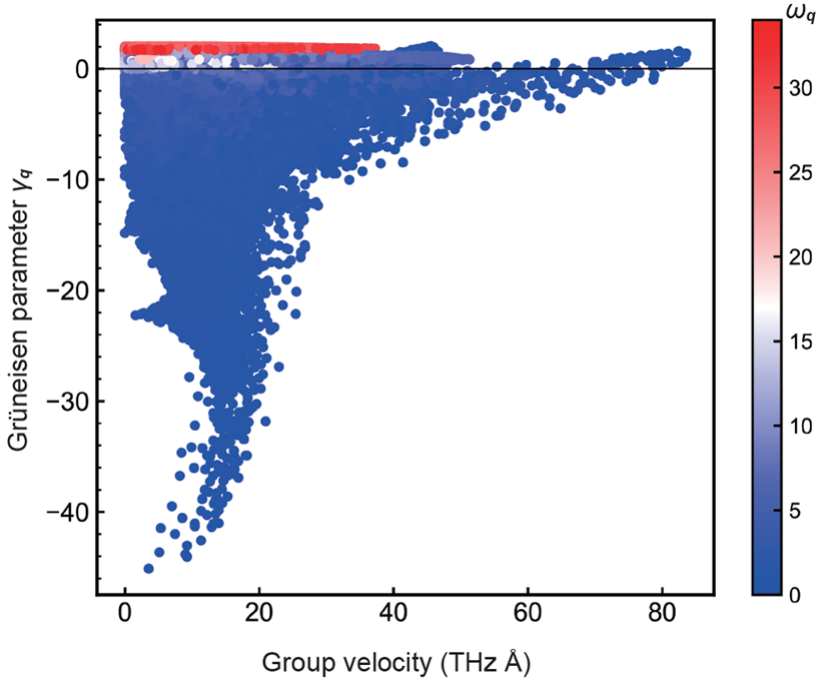
Distribution of the mode-Grüneisen parameters and the group velocity at the relevant wave vectors in the reciprocal space. All of the points are colored with respect to the phonon frequency.

Figure 9 shows the phonon partial densities of states (PDOS) for Cu, P, and 4 symmetrically different O atoms. Here, O1 has the 4e Wyckoff position, while O2, O3, and O4 have the 8f Wyckoff position. In the low-frequency range between 0 and 5 THz, where the mode-Grüneisen parameters are negative, the phonons of Cu and O are dominant. Specifically, the phonon PDOSs of Cu and O4 have large peaks in the *a*- and *c*-axis directions, while those along the *b*-axis direction are relatively small, indicating that Cu and O4 oscillate mainly in the *ac* lattice plane. A peak around 2.5 THz (4 THz) can be seen in the phonon PDOS of O1 projected along the *b*-axis (*c*-axis) connecting the two PO_4_, indicating that O1 oscillates mainly in the *bc* lattice plane.

**Figure 9:**
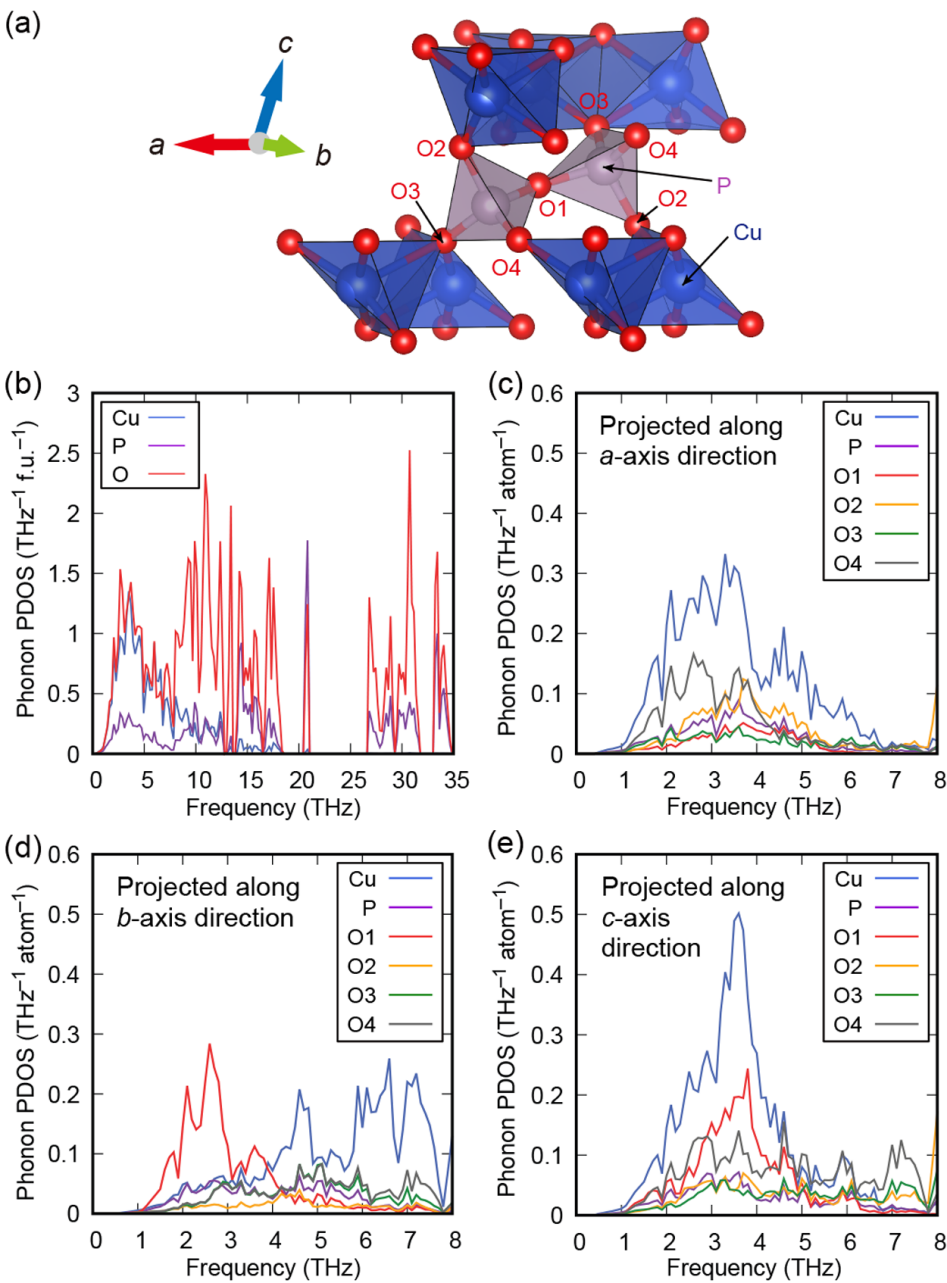
(a) Schematic of Cu_2_P_2_O_7_ with labeled atoms. (b) Phonon partial densities of states (PDOSs) for the relevant atoms. (c–e) Phonon PDOSs for Cu, P, O1, O2, O3, and O4 projected along the *a*-, *b*-, and *c*-axis directions, respectively.

We also show the calculated anisotropic atomic displacement parameter (AADP) and the visualized thermal displacement ellipsoid at 200 K (Figure 10). Note that the relevant atoms are located in the ellipsoids with a probability of 98% (Figure 10b). Our calculation results for AADPs are in considerable agreement with the experimental results (Figure 10a). The largest and second-largest AADPs of oxygen were O1 and O4, respectively, indicating that O1 and O4 largely vibrate compared to O2 and O3. These behaviors can also be observed in phonon PDOS (Figure 9c–e). The AADPs of O1 and O4 are larger than those of O2 and O3 because of the coordination environment difference: O1 and O4 are corner-shared, whereas O2 and O3 are edge-shared. Moreover, from Figure 10b, as discussed above, we can clearly see that the thermal displacement ellipsoid of O1 moves mainly in the *bc* plane, while those of O2, O3, O4, and Cu face the direction perpendicular to CuO_4_ quadrilaterals (*a*- and *c*-axis directions) (see Figure S7). These viewpoints are consistent with the calculated phonon PDOS.

**Figure 10:**
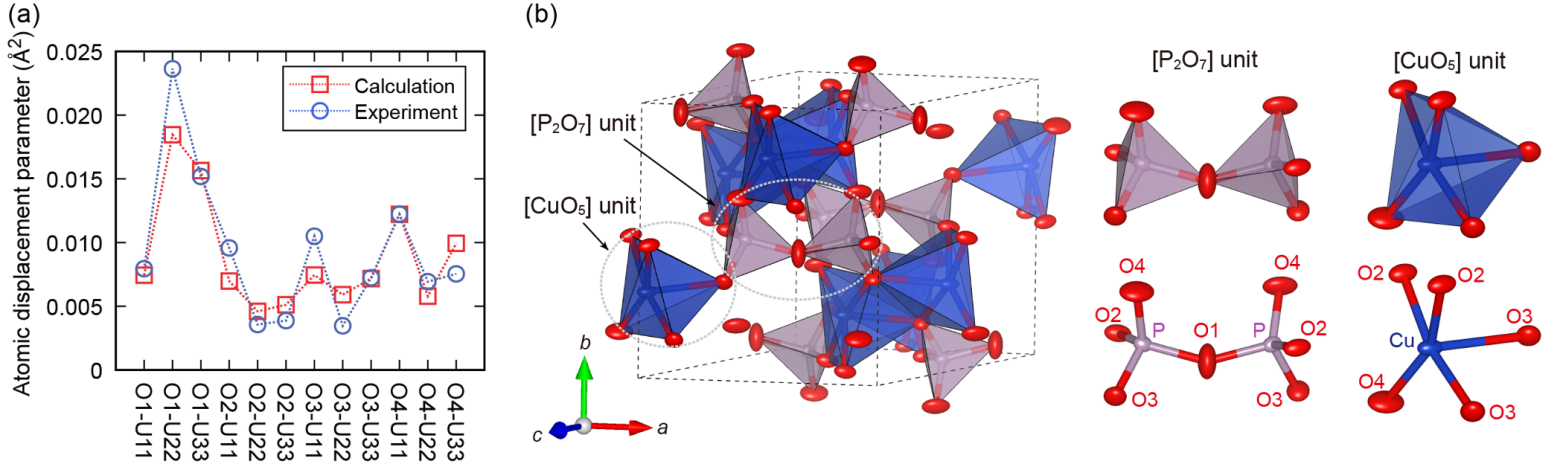
(a) Comparison of calculated and experimental anisotropic atomic displacement parameters of oxygen atoms at 200 K. The experimental values are from ref (12). (b) Structure of Cu_2_P_2_O_7_ with calculated thermal ellipsoids at 200 K representing a 98% probability of containing the relevant atoms.

Considering the phonon DOS at equilibrium volume at the finite temperatures within the QHA and the Bose–Einstein distribution, the phonons in the range between 0 and 7 THz are mainly excited, which have negative mode-Grüneisen parameters (see Figure S8). Therefore, one can infer that the 200 K thermal oscillation ellipsoids and AADPs (Figure 10) reflect the trend of oscillations that contribute mainly to the NTE. On the whole, as illustrated in Figure 10b, O1 oscillates around the P–O1–P bond in the *bc* plane perpendicular to the P–O1–P bond axis, and Cu, O2, and O4 move perpendicular to the bottom of the CuO_5_ pyramidal unit. These vibrational features are important in the NTE behavior because this tendency was also observed in the phonon mode with the most negative mode-Grüneisen parameter (Figure 7).

Finally, we also present the calculation results of COHPs for Cu–O and P–O bonds as shown in Figure 11. One can see that the negative-sign integrated COHP (iCOHP) up to the Fermi level for the Cu–O3 long bond is lower than those for the other Cu–O bonds, indicating that the bond covalency is relatively weak (Figure 11a,b,e,f). In other words, the chemical bonds of Cu and O in the CuO_4_ quadrilaterals (the bottom of the CuO_5_ pyramidal unit) are stronger than those of Cu and O3 in the vertical direction (Cu–O3 long bond). These results should explain why Cu, O2, and O4 move mainly in the direction perpendicular to the bottom of the CuO_5_ pyramidal unit. On the other hand, we can also see that the negative-sign iCOHP up to the Fermi level for the P–O1 bond is lower than those for the other P–O bonds (Figure 11c,d,g,h). These COHP results of P–O bonds could explain why O1 vibrates more drastically than the other oxygens (Figure 10a).

**Figure 11:**
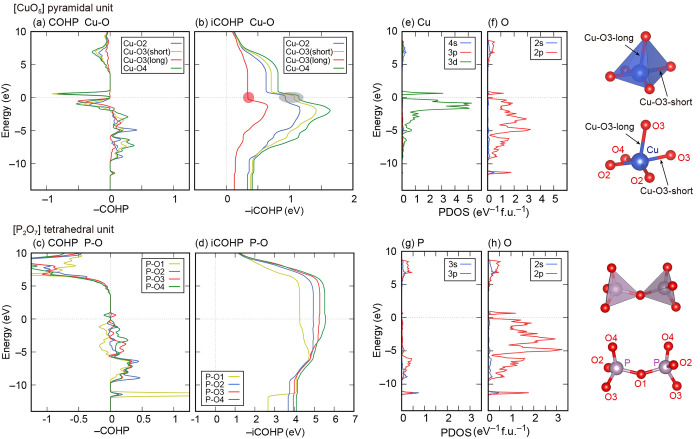
Negative-sign COHPs and iCOHPs for (a, b) Cu–O bondings and (c, d) P–O bondings. (e–h) Projected electronic DOSs of Cu, P, and O. The Cu–O3–long bond connects CuO_4_ plane and apical O3. The Fermi level is set to zero. Atomic schematics of the CuO_5_ pyramidal unit and two PO_4_ tetrahedral units are also illustrated.

In conclusion, by using the first-principles calculations, we investigated the structural stability and analyzed the NTE behavior of monoclinic α-Cu_2_P_2_O_7_, which is a unique material among the typical NTE materials. In the first half, from the investigation of polymorphs and structural stability, we found that small cations *A* and *B* stabilize the α-Cu_2_P_2_O_7_-type structure. The α-Cu_2_P_2_O_7_-type structure is found to be relatively rare, and within the scope of this study, only Cu_2_P_2_O_7_, Cu_2_V_2_O_7_, and Zn_2_V_2_O_7_ were found to be dynamically stable. These results clearly indicate the objective position of Cu_2_P_2_O_7_ in *A*_2_*B*_2_O_7_ polymorphs. In the latter half, to investigate the NTE mechanism of α-Cu_2_P_2_O_7_, we examined the distribution of mode-Grüneisen parameters across the entire first-Brillouin zone. As a result, we found that the phonon mode with the most negative mode-Grüneisen parameter emerged from (^1^/_4_, ^1^/_4_, ^1^/_4_), which is not on the high-symmetry special points. This phonon mode folds the *ac* lattice plane, which should explain the experimental report of Cu_2_P_2_O_7_ showing anisotropic NTE behavior in the *a*- and *c*-axis directions. Our results suggest that the phonon modes should be investigated in the entire first-Brillouin zone to analyze the NTE behavior, particularly for the NTE materials with low space-group symmetry. We believe that our findings will lead to further elucidation of the mechanism of the NTE materials and to the development of new NTE materials.

## Supporting Information Available

Calculated lattice constants of α-Cu_2_P_2_O_7_ within the GGA-PBE, GGA-PBEsol, and meta-GGA-SCAN functionals in the antiferromagnetic configuration (Table S1); cutoff radii and valence electronic configurations of the PAW data sets (Table S2); calculated phonon frequencies at the Γ point of α-Cu_2_P_2_O_7_ and the experimentally reported Raman frequencies (Table S3); schematics of the colinear antiferromagnetic configuration for the *C*2/*c* phase of Cu_2_P_2_O_7_ (Figure S1); comparison of calculated and experimental anisotropic atomic displacement parameters with and without the +*U* correction (Figure S2); calculated phonon bands for α-Cu_2_P_2_O_7_ with and without the +*U* correction (Figure S3); phonon band structures of the *C*2/*c* phases for Cu_2_P_2_O_7_, Cu_2_V_2_O_7_, Zn_2_V_2_O_7_, Cu_2_Ta_2_O_7_, Zn_2_Ta_2_O_7_, Mg_2_Ta_2_O_7_, and Sc_2_Zr_2_O_7_ (Figure S4); ionic radii as a function of the coordination numbers for *A*, *B*, and O (Figure S5); phonon band and distribution of the mode-Grüneisen parameter for the *C*2/*c* phase of Cu_2_P_2_O_7_ on a different band path (Figure S6); schematic of the calculated thermal ellipsoid at 200 K above the *b*-axis (Figure S7); and calculated phonon DOSs and Bose–Einstein distributions (Figure S8) (PDF)
Visualized phonons with negative mode-Grüneisen parameters (ZIP)

## Abbreviations

NTE: negative thermal expansion
QHA: quasi-harmonic approximation
COHPs: crystal orbital Hamilton populations
VBM: valence band maximum
CBM: conduction band minimum
PDOS: partial density of states
AADP: anisotropic atomic displacement parameter
iCOHPs: integrated crystal orbital Hamilton populations
