# Supplementary material for: Mechanism of Negative Thermal Expansion in Monoclinic Cu2P2O7 from First Principles
Source: J Phys Chem Lett. 2023 Dec 27;15(1):156–64. doi: 10.1021/acs.jpclett.3c02856 (PMC10788959; doi:10.1021/acs.jpclett.3c02856)
Supplement: Supplementary file 1 — jz3c02856_si_001.pdf [file jz3c02856_si_001.pdf]

Supporting Information:
Mechanism of Negative Thermal Expansion in Monoclinic $Cu_2P_2O_7$ from First Principles

Yasuhide Mochizuki[†,*], Kaede Nagamatsu[†], Hiroki Koiso, Toshihiro Isobe, and Akira Nakajima

Department of Materials Science and Engineering, School of Materials and Chemical Technology, Tokyo Institute of Technology, Meguro-ku 152-8550, Japan

Email: mochizuki.y.af@m.titech.ac.jp

1. Functional dependencies of lattice constants for $Cu_2P_2O_7$

All of the calculations in the present study have been performed by using the GGA-PBEsol functional. In general, for the lattice dynamics calculations, the degree of consistency of calculated lattice constants with the experimental reports is crucial. To justify the reason why we chose the GGA-PBEsol functional, we have calculated and compared the lattice constants within the GGA-PBEsol and the other widely used functionals (the GGA-PBE and meta-GGA-SCAN functionals) with the experimental reports [S1–S4] as enumerated in Table R1. The calculation results within the GGA-PBEsol functional were in considerably good agreement with the experimental reports [S1–S4]. Indeed, the calculation results within the meta-GGA-SCAN functional were in good agreement with the experimental reports. However, the computational cost of the meta-GGA-SCAN functional is much higher than that of the GGA-PBEsol functional because the energy convergence of the meta-GGA-SCAN functional in self-consistent calculation is poor compared to that of the GGA-PBEsol functional. Therefore, we adopted the GGA-PBEsol functional for all the calculations. We also denote that many computational studies of lattice dynamics and molecular dynamics have reported the calculation results within the PBEsol functional [S5–S8] or the PBE functional [S9–S13].

Table S1. Calculated lattice constants of α-$Cu_2P_2O_7$ within the GGA-PBE, GGA-PBEsol, and meta-GGA-SCAN functionals in the magnetic configuration (AFM2). The experimental reports on the lattice constants [S1–S4] are also enumerated.

	a (Å)	b (Å)	c (Å)	β (deg.)	V (Å^3)
Calc. (PBE)	7.2430	8.2255	9.4221	111.97	520.56
Calc. (PBEsol)	7.0966	8.1179	9.2435	111.28	496.20
Calc. (PBEsol+U5)	7.0062	8.0558	9.2445	110.82	487.71
Calc. (SCAN)	6.9701	8.0903	9.1682	110.31	484.87
Expt. (XRD [S1])	6.876	8.113	9.162	109.54	481.67
Expt. (XRD [S2])	6.895	8.113	9.164	109.62	482.86
Expt. (XRD [S3])	6.887	8.108	9.156	109.54	481.59
Expt. (XRD [S4])	6.901	8.108	9.176	109.65	483.53

2. PAW data sets used in the first-principles calculations

In the present calculations, the PAW data sets enumerated in Table S2 were used with a cutoff energy of 550 eV for the plane-wave basis.

Table S2. Valence electrons and cutoff radii of the PAW data sets used in the present study.

Atom	Valence electrons	PAW cutoff radius (Å)
Mg	$3s^2$	1.52
P	$3s^2 3p^3$	1.23
Sc	$3d^2 4s^1$	1.59
Ti	$3d^3 4s^1$	1.32
V	$3d^4 4s^1$	1.32
Cu	$3d^{10} 4s^1$	1.16
Zn	$3d^{10} 4s^2$	1.27
Ge	$4s^2 4p^2$	1.22
Sr	$4s^2 5p^6 5s^2$	2.14
Y	$4s^2 4p^6 4d^1 5s^2$	1.82
Zr	$4s^2 4p^6 4d^3 5s^1$	1.63
Sn	$5s^2 5p^2$	1.57
Ta	$5d^4 6s^1$	1.50
O	$2s^2 2p^4$	0.82

3. Antiferromagnetic structure for $Cu_2P_2O_7$

As shown in Figure S1, we show the antiferromagnetic configuration used for $Cu_2P_2O_7$. Note that the antiferromagnetic configuration was the most stable among the three possible antiferromagnetic configurations in the primitive $Cu_2P_2O_7$, which is consistent with the previous calculation results [S4, S14].

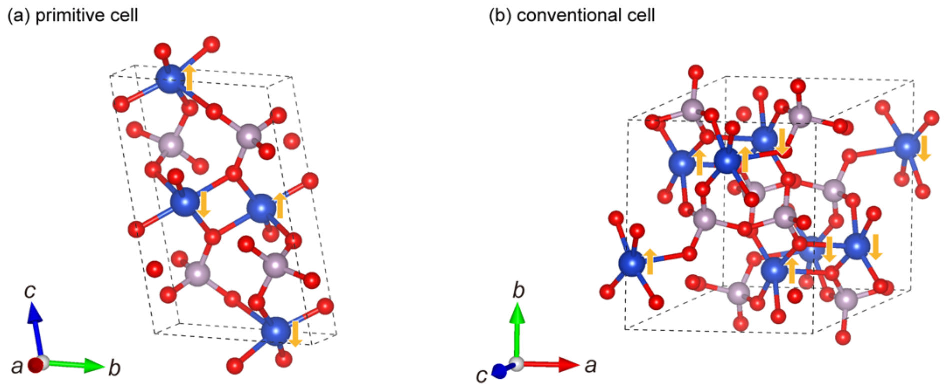

Figure S1. Antiferromagnetic configuration of $Cu_2P_2O_7$ visualized with (a) the primitive cell and (b) the conventional cell. Yellow arrows indicate the direction of the magnetic moment in Cu.

4. The correlation effect owing to +*U* correction for the *d* electrons in Cu^{2+} ions

The formal charge of Cu in $Cu_2P_2O_7$ is 2+, which gives a d^9 electronic configuration for Cu ions. The electrons correlation effect of d^9 in Cu^{2+} has been extensively studied since the discovery of high-temperature cuprate superconductors. In the field of strongly correlated electrons systems, it is widely known that a strong electronic correlation generally gives a localization effect to the electrons, leading a system to a Mott insulator and/or a magnetic ordering [S15]. However, our calculation results of $Cu_2P_2O_7$ have clearly shown that the magnetic moments and antiferromagnetic structure remain even without +*U* correction, indicating that the +*U* correction in 3*d* electrons in Cu^{2+} ions is not necessarily essential. This is probably due to the superexchange interaction of Cu_2O_6 magnetic dimers [S16]. In short, considering the magnetic structure is much more important than the +*U* correction. To show the validity of our insistence, we also calculated the anisotropic atomic displacement parameters of oxygen atoms at 200 K with and without the +*U* correction, as shown in Figure S2. Here, the Dudarev formulation was adopted for the +*U* correction [S17]. From these results, little change can be observed by applying the +*U* correction.

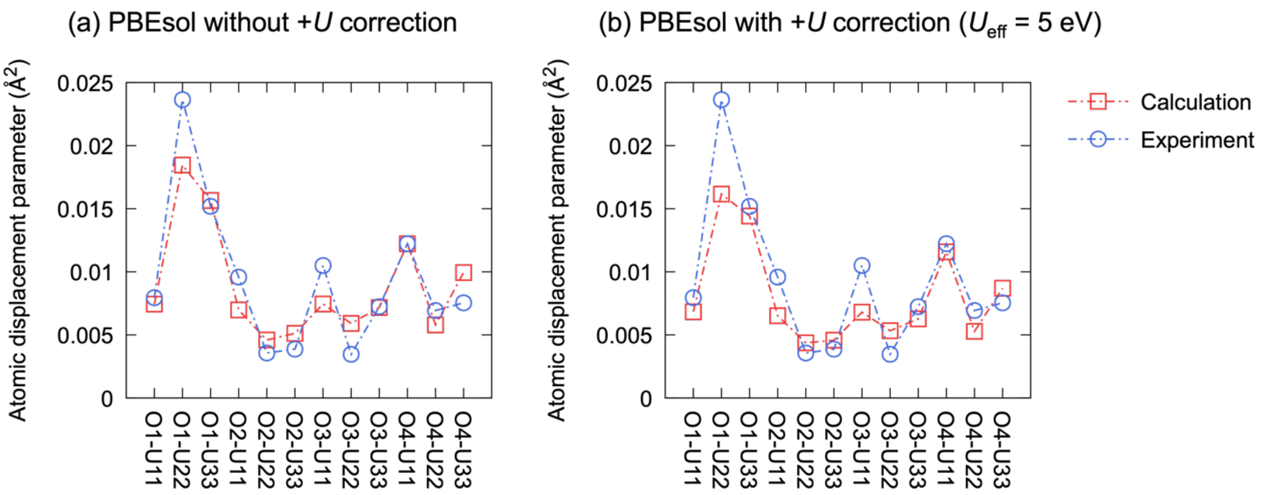

Figure S2. Comparison of calculated and experimental anisotropic atomic displacement parameters of oxygen atoms at 200 K (a) without and (b) with +*U* correction.

In addition, we also show the calculated phonon bands with and without the +*U* correction for the 3*d* electrons in Cu, as illustrated in Figure S3. The little change in the low-frequency region owing to the +*U* correction can be observed, while the increase of phonon frequency in the high-frequency region can also be observed. The increase of phonon frequency should stem from the shrinkage of lattice constants due to the +*U* correction (compare the calculated lattice constants of PBEsol and PBEsol+*U*5 in Table S1). Furthermore, the atomic displacement parameters are largely affected mainly by the low-frequency phonons, which can explain the reason why the +*U* correction gives little change in them (Figure S2).

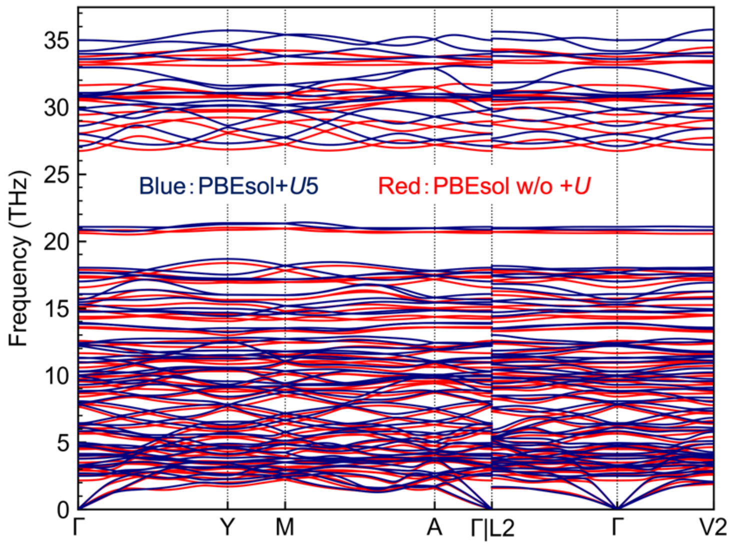

Figure S3. Calculated phonon band structures for α-$Cu_2P_2O_7$ with (blue line) and without +*U* correction (red line).

5. The determination of the most stable phase in the comparison of polymorphs

For the comparison of polymorphs for $A_2B_2O_7$ (A = Cu, Zn, Mg, Sr, Sc, Y; B = Ge, Sn, Ti, Zr, P, V, Ta), the total energies of the $C2/c$ and $C2/m$ phases for $Mg_2P_2O_7$, $Sc_2Ti_2O_7$, and $Mg_2Ta_2O_7$ are comparable. Therefore, we determined the most stable phase of these three compounds as follows: as the most stable phase, we adopted the $C2/m$ phase for $Mg_2P_2O_7$, which is experimentally reported [S18], and the $C2/c$ ($C2/m$) phase for $Mg_2Ta_2O_7$ ($Sc_2Ti_2O_7$) because the total energy was slightly lower than that of the $C2/m$ ($C2/c$) phase.

We also present the phonon bands of the $Cu_2P_2O_7$-type $C2/c$ phases for $Cu_2P_2O_7$, $Cu_2V_2O_7$, $Zn_2V_2O_7$, $Sc_2Zr_2O_7$, $Cu_2Ta_2O_7$, $Zn_2Ta_2O_7$, and $Mg_2Ta_2O_7$ as shown in Figure S4. We can see that the three $C2/c$ phases of $Cu_2P_2O_7$, $Cu_2V_2O_7$, and $Zn_2V_2O_7$ are found to be dynamically stable [Figure S4a–c], whereas the other four $C2/c$ phases of $Sc_2Zr_2O_7$, $Cu_2Ta_2O_7$, $Zn_2Ta_2O_7$, and $Mg_2Ta_2O_7$ are found to have imaginary phonon modes, indicating that they are dynamically unstable [Figure S4d–g].

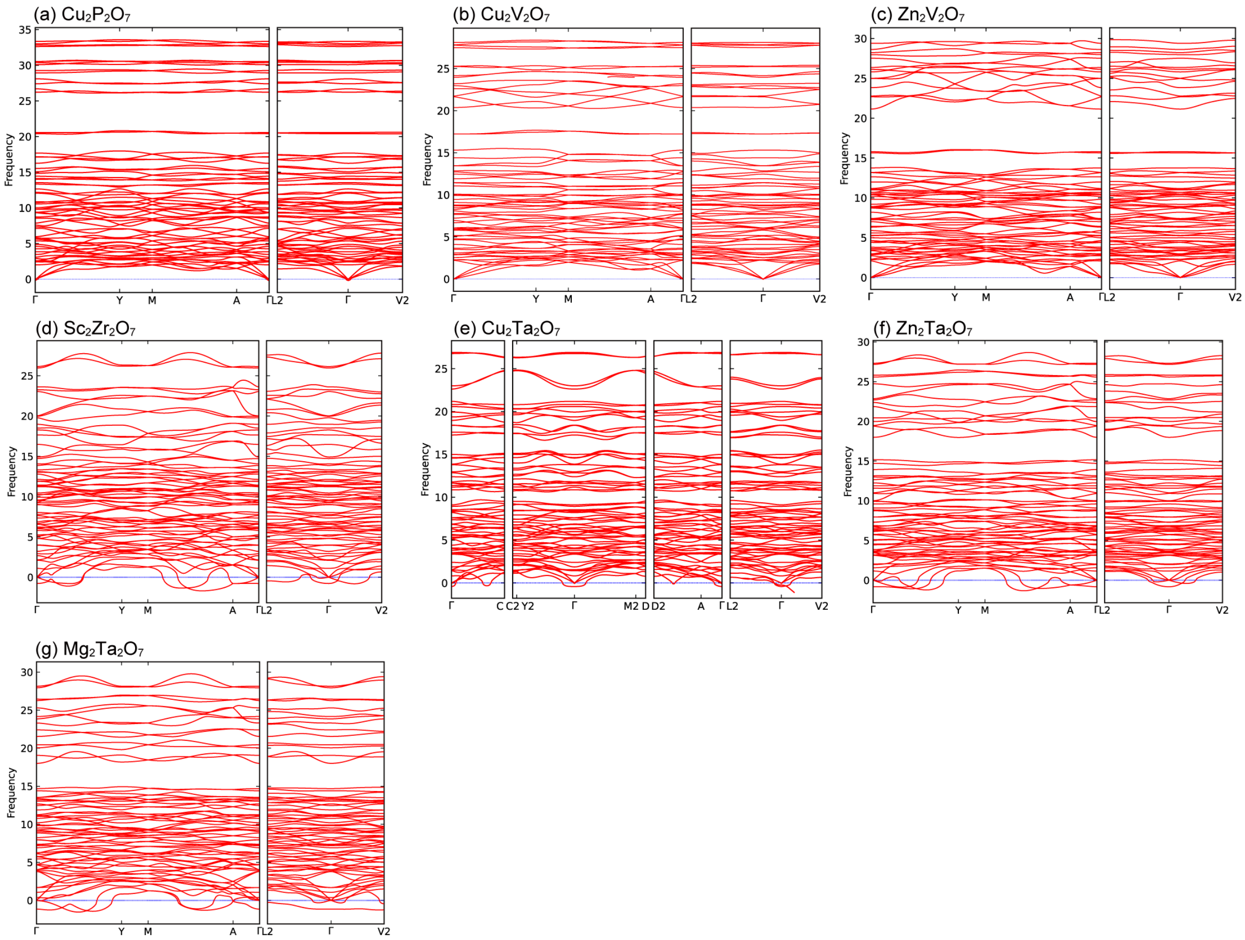

Figure S4. Phonon dispersion curves of (a) $Cu_2P_2O_7$, (b) $Cu_2V_2O_7$, (c) $Zn_2V_2O_7$, (d) $Sc_2Zr_2O_7$, (e) $Cu_2Ta_2O_7$, (f) $Zn_2Ta_2O_7$, and (g) $Mg_2Ta_2O_7$ in the $Cu_2P_2O_7$-type $C2/c$ phases.

6. The cationic radii for the comparison of polymorphs

In the map of structural stability (Figure 3), we evaluated the cationic radii of *A* and *B* by using Shannon's ionic radii [S19]. We present the coordination number dependence of ionic radius as shown in Figure S3. Here, we linearly approximated the correlation between the coordination number and ionic radius, and then we evaluated the cationic radii *A* and *B* for the relevant polymorphs by using the linear regression model and the effective coordination number extracted by using VESTA [S20].

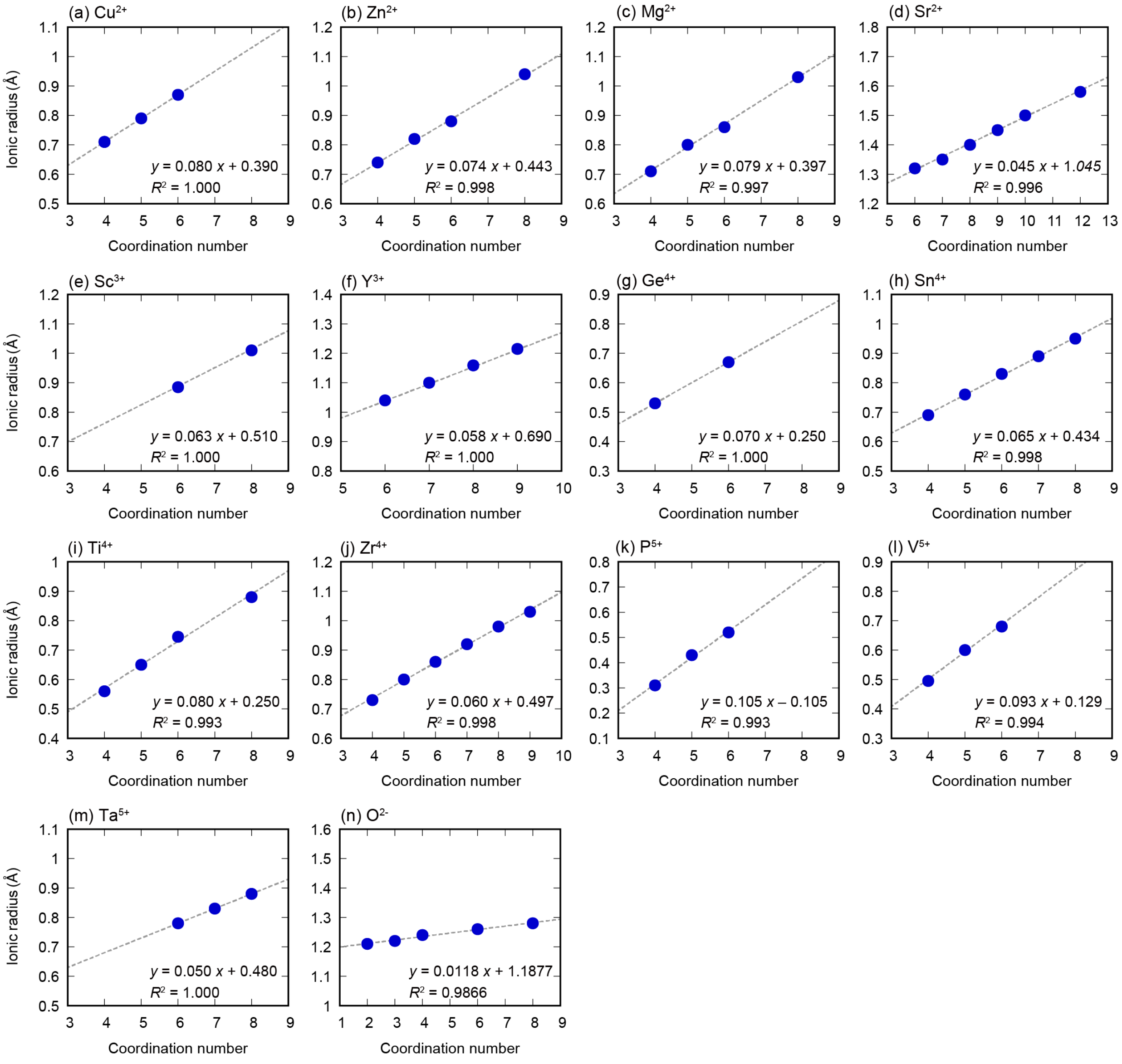

Figure S5. Coordination number dependences of ionic radii for cations *A*, *B* and O. The dashed lines are the least-squares linear fittings, and R^2 values denote their coefficients of determination.

7. Comparison of calculated phonon frequencies with the experimental reports

To show the validity of the calculated phonon frequencies in the present study, we compared them with the experimental reports. The calculated phonon frequencies at Γ point and experimentally reported Raman frequencies are enumerated in Table S3. There are 3 acoustic and 63 optic modes; the phonon modes that are active to Raman and IR spectroscopy are 16 A_g + 17 B_g and 15 A_u + 15 B_u, respectively. One can see that our calculation results are compatible with the experimental reports. The little difference in phonon frequencies between the calculation results and experimental reports is owing to the difference in lattice constants (see Table S1).

Table S3. Calculated phonon frequency at Γ point of $Cu_2P_2O_7$ and the experimentally reported Raman frequencies [S21] in the unit of cm^{-1}.

	IR active mode		Raman active mode	
Irreducible representation	A_u	B_u	A_g	B_g
Calculation	86, 100, 194, 204, 255, 323, 367, 419, 451, 474, 576, 693, 935, 1017, 1113	100, 154, 159, 261, 304, 333, 365, 388, 479, 509, 596, 891, 982, 1019, 1113	72, 97, 134, 188, 202, 289, 318, 334, 408, 454, 496, 580, 687, 965, 1025, 1106	103, 116, 127, 150, 208, 258, 280, 324, 353, 410, 504, 525, 552, 918, 1021, 1055, 1126
Experiment [S21]	N/A		105, 121, 135, 141,160, 214, 219, 282, 320, 360, 412, 475, 544, 590, 730, 975, 1050, 1080, 1146, 1217	

8. The distribution of mode-Grüneisen parameter on a different band path

As shown in Figure S6, we show the phonon band and the distribution of mode-Grüneisen parameters for the *C*2/*c* phases of $Cu_2P_2O_7$ along with the band path suggested by Hinuma *et al* [S22]. In Figure 5, the band path passes the reciprocal point (1/4, 1/4, 1/4), whereas the band path suggested by Hinuma *et al* does not (Figure S6). These results indicate that the selection of band paths might miss the significant phonon modes for the NTE behavior.

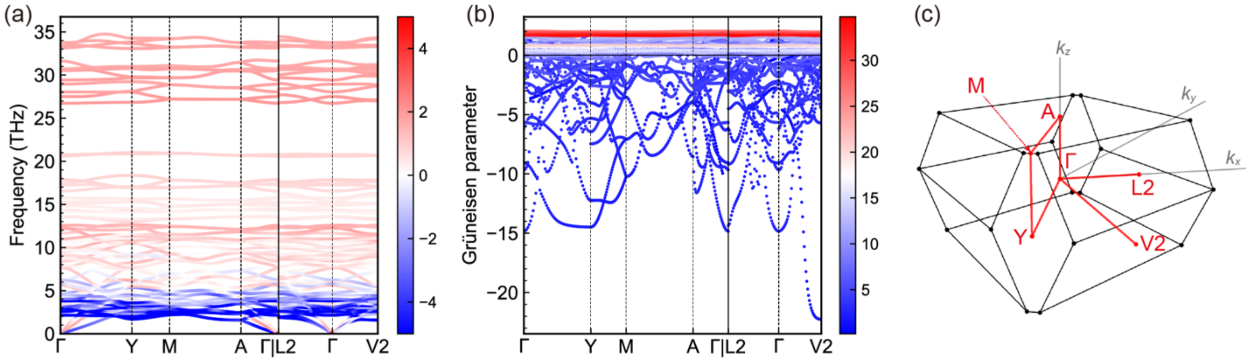

Figure S6. (a) Phonon band structure with respect to the values of the mode-Grüneisen parameters for $Cu_2P_2O_7$. Blue and red indicate negative and positive values of mode-Grüneisen parameters, respectively. (b) Mode-Grüneisen parameters along the path of first-Brillouin zone of *C*2/*c* space group, which are colored according to the calculated phonon frequency. (c) First Brillouin zone shape and *k*-paths generated from the Hinuma *et al* [S22].

9. Thermal ellipsoids of $Cu_2P_2O_7$ at 200 K visualized above the *b*-axis

Figure S7 shows the structure of $Cu_2P_2O_7$ with calculated thermal ellipsoids at 200 K visualized above the *b*-axis. Note that the relevant atoms are located in the ellipsoids with a probability of 98%. We can observe that the O2, O3, O4, and Cu atoms move in the direction perpendicular to CuO_4 quadrilaterals.

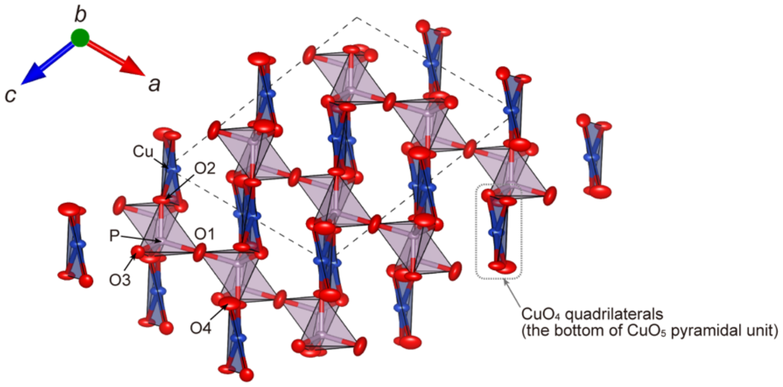

Figure S7. Schematic of $Cu_2P_2O_7$ with calculated thermal ellipsoids at 200 K representing a 98% probability of containing the relevant atoms above the *b*-axis. PO_4 tetrahedral units and CuO_4 quadrilaterals are illustrated.

10. The number of excited phonons in the finite temperatures

Figure S8 shows the calculated total phonon densities of states and the Bose-Einstein distributions at 100, 200, and 300 K. Here, we adopted the equilibrium volumes at the relevant finite temperatures obtained by minimizing free energy via QHA. In the finite temperatures, the phonons in the range between 0 and 7 THz are mainly excited, which have negative mode-Grüneisen parameters (Figure 5a).

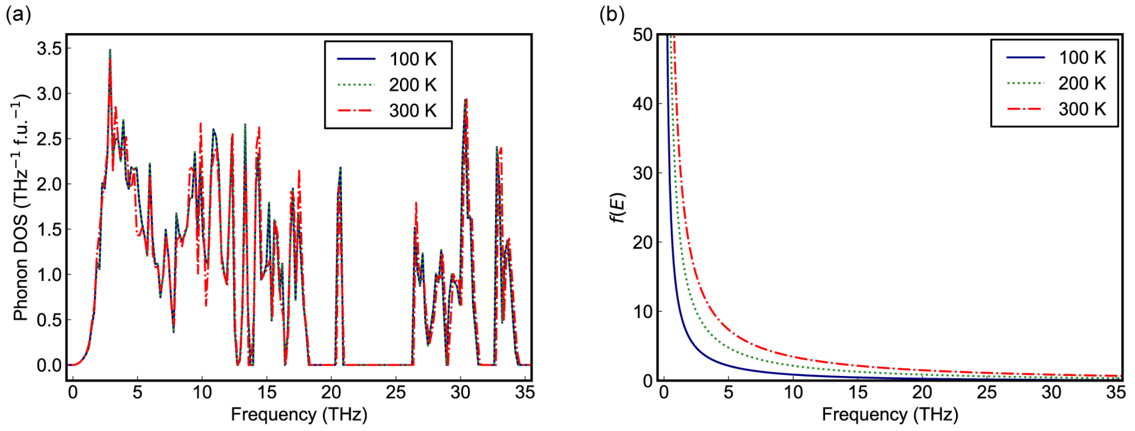

Figure S8. (a) Calculated total phonon densities of states and (b) the Bose-Einstein distributions at 100 K, 200 K, and 300 K.

REFERENCES

[S1] Robertson, B. E.; Calvo, C. The crystal structure and phase transformation of α-$Cu_2P_2O_7$. *Acta Cryst.* **1967**, *22*, 665.

[S2] Effenberger, H. Structural refinement of low-temperature copper(II) pyrophosphate. *Acta Cryst. C* **1990**, *46*, 691.

[S3] Le, S.-N.; Navrotsky, A.; Pralong, V. Energetics of copper diphosphates – $Cu_2P_2O_7$ and $Cu_3(P_2O_6OH)_2$. *Solid State Sci.* **2008**, *10*, 761.

[S4] Pastukh, S.; Laskowska, M.; Dulski, M.; Krzykawski, T.; Parlinski, K.; Piekarz, P. *Ab initio* studies for characterization and identification of nanocrystalline copper pyrophosphate confined in mesoporous silica. *Nanotechnology* **2021**, *32*, 415701.

[S5] He, R.; Wu, H.; Lu, Y.; Zhong, Z. Origin of negative thermal expansion and pressure-induced amorphization in zirconium tungstate from a machine-learning potential. *Phys. Rev. B* **2022**, *106*, 174101.

[S6] Ritz, E. T.; Li, S. J.; Benedek, N. A. Thermal expansion in insulating solids from first principles. *J. Appl. Phys.* **2019**, *126*, 171102.

[S7] Ritz, E. T.; Benedek, N. A. Interplay between Phonons and Anisotropic Elasticity Drives Negative Thermal Expansion in $PbTiO_3$. *Phys. Rev. Lett.* **2018**, *121*, 255901.

[S8] Ablitt, C.; Craddock, S.; Senn, M. S.; Mostofi, A. A.; Bristowe, N. C. The origin of uniaxial negative thermal expansion in layered perovskites. *npj Comput. Mater.* **2017**, *3*, 44.

[S9] Wang, L.; Chen, Y.; Ni, J.; Ye, F.; Wang, W. Anharmonic Interaction in Negative Thermal Expansion Material $CaTiF_6$. *Inorg. Chem.* **2022**, *61*, 17378.

[S10] Gupta, M. K.; Chaplot, S. L. Negative Thermal expansion in cubic ZrW_2O_8: Role of phonons in the entire Brillouin Zone from *ab Initio* calculations. *Phys. Rev. B* **2013**, *88*, 014303.

[S11] Wang, Z.; Wang, F.; Wang, L.; Jia, Y.; Sun, Q. First-principles study of negative thermal expansion in zinc oxide. *J. Appl. Phys.* **2013**, *114*, 063508.

[S12] Dove, M. T.; Wei, Z.; Phillips, A. E.; Keen, D. A.; Refson, K. Which phonons contribute most to negative thermal expansion in ScF3? *APL Mater.* **2023**, *11*, 041130.

[S13] d'Ambrumenil, S.; Zbiri, M.; Chippindale, A. M.; Hibble, S. J.; Marelli, E.; Hannon, A. C. Lattice dynamics and nega-tive thermal expansion in the framework compound $ZnNi(CN)_4$ with two-dimensional and three-dimensional local environments. *Phys. Rev. B* **2019**, *99*, 024309.

[S14] Yang, X., Zhang, P.; Korzhavyi, P. Hybrid-Density Functional Calculations of Structural, Electronic, Magnetic, and Thermodynamic Properties of α-$Cu_2P_2O_7$. *Appl. Sci.* **2023**, *13*, 498.

[S15] Imada, M.; Fujimori, A.; Tokura, Y. Metal-Insulator Transitions. *Rev. Mod. Phys.* **1998**, *70*, 1039.

[S16] Janson, O.; Tsirlin, A. A.; J. Sichelschmidt; Y. Skourski; Weickert, F.; H. Rösner. Long-range superexchange in $Cu_2A_2O_7$. *Phys. Rev. B* **2011**, *83*, 094435.

[S17] Dudarev, S. L.; Botton, G. A.; Savrasov, S. Y.; Humphreys, C. J.; Sutton, A. P. Electron-energy-loss spectra and the structural stability of nickel oxide: An LSDA+*U* study. *Phys. Rev. B* **1998**, *57*, 1505.

[S18] Calvo, C. Refinement of the crystal structure of β-$Mg_2P_2O_7$. *Canadian J. Chem.* **1965**, *43*, 1139.

[S19] Shannon, R. D. Revised Effective Ionic Radii and Systematic Studies of Interatomie Distances in Halides and Chaleogenides. *Acta Cryst.* **1976**, *32*, 751.

[S20] Momma, K.; Izumi, F. VESTA 3 for three-dimensional visualization of crystal, *J. Appl. Crystallogr.* **2011**, *44*, 1272.

[S21] Pogorzelec-Glaser, K.; Pietraszko, A.; Hilczer, B.; Połomska, M. Structure and phase transitions in $Cu_2P_2O_7$. *Phase Transitions* **2006**, *79*, 535.

[S22] Hinuma, Y.; Pizzi, G.; Kumagai, Y.; Oba, F.; Tanaka, I. Band structure diagram paths based on crystallography. *Comput. Mater. Sci.* **2017**, *128*, 140.
